# Increased Expression of PITX2 Transcription Factor Contributes to Ovarian Cancer Progression

**DOI:** 10.1371/journal.pone.0037076

**Published:** 2012-05-15

**Authors:** Frederic K. C. Fung, David W. Chan, Vincent W. S. Liu, Thomas H. Y. Leung, Annie N. Y. Cheung, Hextan Y. S. Ngan

**Affiliations:** 1 Department of Obstetrics and Gynaecology, LKS Faculty of Medicine, The University of Hong Kong, Hong Kong, Special Administrative Region, People's Republic of China; 2 Department of Pathology, LKS Faculty of Medicine, The University of Hong Kong, Hong Kong, Special Administrative Region, People's Republic of China; Cedars-Sinai Medical Center, United States of America

## Abstract

**Background:**

Paired-like homeodomain 2 (PITX2) is a bicoid homeodomain transcription factor which plays an essential role in maintaining embryonic left-right asymmetry during vertebrate embryogenesis. However, emerging evidence suggests that the aberrant upregulation of PITX2 may be associated with tumor progression, yet the functional role that PITX2 plays in tumorigenesis remains unknown.

**Principal Findings:**

Using real-time quantitative RT-PCR (Q-PCR), Western blot and immunohistochemical (IHC) analyses, we demonstrated that PITX2 was frequently overexpressed in ovarian cancer samples and cell lines. Clinicopathological correlation showed that the upregulated PITX2 was significantly associated with high-grade (*P* = 0.023) and clear cell subtype (*P* = 0.011) using Q-PCR and high-grade (*P*<0.001) ovarian cancer by IHC analysis. Functionally, enforced expression of PITX2 could promote ovarian cancer cell proliferation, anchorage-independent growth ability, migration/invasion and tumor growth in xenograft model mice. Moreover, enforced expression of PITX2 elevated the cell cycle regulatory proteins such as Cyclin-D1 and C-myc. Conversely, RNAi mediated knockdown of PITX2 in PITX2-high expressing ovarian cancer cells had the opposite effect.

**Conclusion:**

Our findings suggest that the increased expression PITX2 is involved in ovarian cancer progression through promoting cell growth and cell migration/invasion. Thus, targeting PITX2 may serve as a potential therapeutic modality in the management of high-grade ovarian tumor.

## Introduction

Ovarian cancer is one of the most common cancer among the female population in worldwide [1].The high mortality rate of ovarian cancer is due to the late diagnosis and poor therapeutic response because the disease is often manifested with little or non-specific symptoms at the early stage [2], [3], [4]. Ovarian cancer can be classified into four subtypes; serous, endometrioid, mucinous and clear cell, based on the histological differentiation of ovarian epithelium and underlying genetic alterations [3], [5], [6]. The grading of ovarian tumor is categorized in accordance with International Federation of Gynaecology and Obstetrics (FIGO) system, revealing that high-grade tumor exhibit the characteristic of faster cell growth and poor prognosis as well as chemoresistance compared with low grade tumor [7], [8].

Paired-like homeodomain 2 (PITX2) transcription factor is the member of bicoid homeodomain family and plays an important role in embryogenesis of vertebrates [9]. Several studies have reported that mutation of PITX2 is associated with the pathogenesis of Axenfeld-Rieger syndrome (ARS) which is an autosomal dominant human disease characterized by developmental defects of eye, teeth and heart [6]. Recent reports have documented that PITX2 is overexpressed in nonfunctional pituitary adenomas [10], node-positive colorectal cancer [11] and thyroid cancer [12]. Moreover, inhibition of PITX2 expression by shRNA in thyroid cancer cells significantly reduced the capacity of cell growth in soft-agar assay, suggesting that PITX2 may have oncogenic potential and may be involved in tumor progression [12].

In the present study, we demonstrated that PITX2 was frequently upregulated and was significantly associated with high-grade ovarian cancer. Using ovarian cancer cell models, we revealed that the upregulated PITX2 could elevate cell cycle regulators such as CyclinD1 and C-myc, and promote cell proliferation, cell migration/invasion as well as tumor growth in xenograft model mice. Our findings provide further insight for the oncogenic role of PITX2 in mediating ovarian cancer tumorigenesis.

## Materials and Methods

### Clinical samples and cell lines

Surgical resection of 97 tumor samples from primary ovarian cancer patients and normal ovaries samples from benign disease were randomly chosen for Q-PCR analysis. The histological subtype and stage of the tumors were categorized according to International Federation of Gynaecology and Obstetrics (FIGO) classification. Written informed consent was taken by the above participants and the use of these clinical samples was approved by Institutional Review Board of the University of Hong Kong/Hospital Authority Hong Kong West Cluster (HKU/HA HKW IRB)(Institutional Review Board number: UW05-143 T1806). Two immortalized human ovarian surface epithelial cells were used: HOSE 10-2 and HOSE 17-1(kindly provided by Professor George Tsao, The University of Hong Kong) [13]. An immortalized human oviductal epithelial cell line, OE-E6/E7, was obtained from Dr. Calvin Lee (The University of Hong Kong) [14]. Nine ovarian cancer cell lines were used: OV2008, C13*, A2780s, A2780cp (kindly provided by Professor Benjamin Tsang, The University of Ottawa) [13], [15], OV420, OV429, OV433, OVCAR3, SKOV-3, TOV21G, mouse fibroblast Wnt3a L cells and Human Embryonic Kidney 293 cells (HEK 293) (America Type Culture Collection, Rockville, MD, USA). All cell lines were cultured in either minimum essential medium or Dulbecco's modified Eagle medium with 10% fetal bovine serum and 1% Penicillin-Streptomycin in 75 cm^2^ flasks and incubated at 37°C in 5% CO_2_.

### Plasmids and cell transfection

pCI-*HA-PITX2A* expressing plasmid (gift from Dr. Kathy Kozlowski, University of Michigan) was used for ectopic expression of HA-tagged PITX2A. The plasmid contains full length human *PITX2A*cDNA and its expression was driven by the CMV promoter. The pCI vector was used as the negative control. The HuSHpGFP-V-RS plasmid vector and short hairpin RNA interference (shRNA) targeting *PITX2* in pGFP-V-RS vector (pGFP-V-RS-*PITX2*) were purchased from OriGene Technologies (OriGene Technologies, Inc, Rockville, MD, USA). The shRNA sequence targeting *PITX2* was 5′ GCC GTT GAA TGT CTC TTC TCC AAA GAC TC 3′.Lipofectamine™ 2000 (Invitrogen Life Technologies, Carlsbad, CA, USA) was used for cell transfection according to the manufacturer's instructions. Stable cells overexpressing PITX2A or knockdown of PITX2 were harvested after 14 days of puromycin (1 µg/ml) selection and verified by Western blot analysis.

### RNA extraction and reverse transcriptase-PCR

Total RNA from the clinical samples and the cultured cells was isolated using the TRIzol reagent (Invitrogen). The cDNA was prepared using Reverse transcription reagent kit (Applied Biosystems, Foster City, CA, USA). The quantitative reverse transcriptase-PCR (Q-PCR) used for evaluation the expressions of *PITX2*, *Cyclin-D1* and *C-myc* was performed by Taqman® Gene expression Assays; human *PITX2* (Assay ID: Hs00165626_m1), human *Cyclin-D1* (Assay ID: Hs00765553_m1) and human *C-myc* (Assay ID: Hs00153408_m1), in an ABI PRISM™ 7500 system (Applied Biosystems). The human *18S rRNA* (Assay ID: Hs99999901_m1) was used as an internal control.

### Western blot analysis

Cells were lysed by lysis buffer (Cell Signaling Technology) containing protease inhibitor (Sigma) and Phenylmethylsulfonyl fluoride (PMSF) (Sigma Chemical Co., St Louis, MO, USA). The protein samples were separated by 10% SDS-PAGE and electroblotted onto the Hybond-P membranes (Amersham Pharmacia Biotech, Cleveland, OH, USA). Blots were blocked with 5% skim milk, followed by incubation with PITX2 (C16) and C-Myc (N262) (Santa Cruz Biotechnology, Inc., Santa Cruz, CA, USA), Cyclin-D1 and β-Catenin (Cell Signaling), Anti-HA and β-actin(Sigma) overnight at 4°C. Blots were then incubated with anti-mouse, anti-rabbit (Amersham Pharmacia Biotechnology) and anti-goat (Santa Cruz) secondary antibodies conjugate with horseradish peroxidase for 1 hour in room temperature and visualized using ECL™ Western Blotting Detection Reagent (Amersham).

For immunohistochemical analysis, two commercial ovarian cancer tissue arrays (OVC1021 and OVC481, Pantomics Inc, San Francisco, CA)was immunostained with primary rabbit polyclonal anti-PITX2 antibody (AbcamInc, Cambridge, MA, USA) in 1∶200 dilution. The stained section was identified as positive or negative. For those immuno-positive samples, the intensity of the staining (+1, faint, +2 moderate, +3 strong and +4 very strong) and the proportion of stained area (0–100%) were scored. The immunoreactivity of each sample was determined by multiplying the intensity and percentage of stained area. The mean of immunoreactivity value of normal and borderline cases was used to normalize all cases. All tissue section was examined and scored independently by two investigators.

### Cell viability assay

Cell viability was analyzed by cell proliferation kit (XTT) (Roche Applied Science, Indianapolis, IN, USA) according to the manufacturer's instruction. Each sample was performed triplicate and three independent experiments were carried out.

### Soft-agar assay

A total of 1×10^4^ cells were prepared in 1.5 ml full medium containing 0.6% agarose. The mixtures were added onto the solidified bottom layer containing 1% agar in 2 ml full medium. Viable colonies were counted and photographed after 14–36 days. The experiment were performed in triplicate and carried out three times independently.

### Wound Healing assay

Cells were seeded in a six well plate until it reached full confluence in a monolayer. Next, medium in each well was replaced by fresh medium containing Mitomycin C (10 µg/ml) (Sigma) and incubated for 3 hours at 37°C.A single wound was created in the middle of each well using a micro-pipette tip. The plate was incubated at 37°C at 5% CO_2_. The image of wound closure was taken at different time courses. The relative migration rate was expressed as relative width of the wounds/time. Three independent experiments were performed in triplicate.

### Transwell cell migration and invasion assay

Quantification of cell migration and invasion were performed using QCM™ 24-Well Colorimetric Cell Migration Assay (Millipore Corporation, Billerica, MA, USA) and Cell Invasion Assay Kit (Chemicon International, Temecula, CA, USA) according to the manufacturer's instruction. Three different fields of the stained cells were photographed and counted for each wells. The experiments were performed three times independently.

### *In vivo* tumor xenograft model

To investigate whether PITX2 overexpression promotes tumor growth *in vivo*, PITX2 stably overexpressed SKOV-3 cells were injected subcutaneously into nude mice. Diameter of the tumor were measured every three days and the mice were scarified a month after the injection. The tumor volume was calculated as (mean of diameters)^3^×π/6. All animal experiments were approved by the University of Hong Kong Committee on the Use of Live Animals in Teaching and Research (CULATR No. 2053-09).

### Data analysis

Data were presented as mean ± SD. Student's *t*-test was used to analysis parametric data. A *P*-value of <0.05 was considered significant in all experiment.

## Results

### UpregulatedPITX2 is observed in ovarian cancer cells

**Figure 1:**
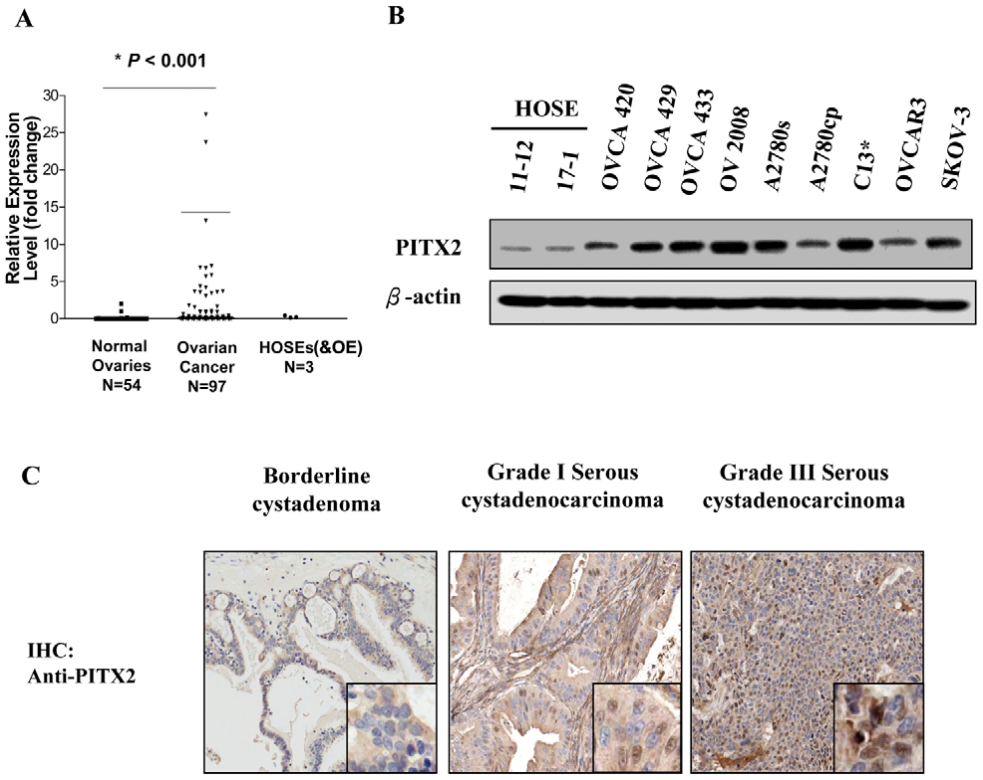
*PITX2* is upregulated in ovarian cancer samples and cell lines. (**A**) Quantitative RT-PCR analysis was performed in normal ovaries (n = 54) and ovarian cancer samples (n = 97) using *PITX2* specific primer. 18S and TATA-box binding protein (TBP) were used as the internal loading controls. *P<0.001. (**B**) Western blot analysis using anti-PITX2 antibody to evaluate the expression level of PITX2 (isoforms A, B and C)) (35 kDa) in ovarian cancer cell lines (n = 9) and HOSE cell lines (n = 2). β-actin was used as a loading control. (**C**) Immunohistochemical analysis of PITX2 expression (nuclear staining) in borderline cystadenoma and high-grade (3) serous cystadenocarcinoma on an ovarian cancer tissue array (OVC1021). Magnification: 20×.

Despite the functions of PITX2 during embryogenesis have been extensively studied, the functional roles of PITX2 in human cancers remain largely unknown. Here, we evaluated the expression status of *PITX2* in ovarian cancer samples (n = 97), normal ovaries (n = 54), normal ovarian cell lines including two HOSEs (HOSE 10-2 and HOSE 17-1) and one immortalized human oviductal epithelial cell line (OE-E6/E7) by Q-PCR. We found that *PITX2* was significantly upregulated in ovarian cancer samples (∼15 folds) as compared with normal ovaries and ovarian cell lines (*P*<0.001) (Figure 1A). Clinicopathological correlation showed that the overexpressed *PITX2* was remarkably associated with high-grade (grade 3) (*P* = 0.023) and clear cell subtype ovarian cancer (*P* = 0.011) (Table 1). However, there was no significant association between the overexpressed *PITX2* and other parameters such as age, stage and recurrence (Table 1). Besides, Western blot analysis was also conducted to compare the expression of PITX2 in a panel of human ovarian cancer cell lines and normal ovarian surface epithelium (HOSEs) cells. Compared with HOSEs cell lines, a widespread increase in PITX2 expression was observed in all ovarian cancer cell lines (Figure 1B). In addition, our Western blot data also showed that PITX2 was obviously upregulated in a clear cell subtype ovarian cancer cell line (TOV21G) when compared to OE-E6/E7 (Figure S1A), the immortalized normal fallopian tube epithelial cell line. This result further confirms the above clinicopathological findings. To further evaluate the protein level of PITX2 in ovarian cancer samples, immunohistochemical staining of PITX2 was performed in an ovarian cancer tissue array (OVC481) which includes 16 cases of ovarian cancer samples paired with normal ovaries. Consistent with the Q-PCR findings, increased expression of PITX2 was usually observed in ovarian cancer samples particularly in high-grade or undifferentiated tumors as compared with their corresponding uninvolved normal ovarian tissues (Figure S1B). Furthermore, we examined the expression of PITX2 in a bigger pool of ovarian cancer samples by using another commercial ovarian cancer tissue array (OVC1021) which includes 2 normal, 2 benign, 1 borderline cystademoma and 97 malignant tumor samples. Our finding showed that the upregulated PITX2 was significantly correlated with high-grade ovarian tumor only (*P*<0.001) and was consistent with the result of Q-PCR analysis (Table 2).

**Table 1:** PITX2 expression and clinicopathological co-relation in ovarian cancer.

		PITX2 expression level		
Characteristics	Total	< = 3 folds	>3 folds	*P-value*
**All cases**	78	30(38.46%)	48(61.54%)	
**Age (y)**				
<50	42	16(38.1%)	26(61.9%)	
≧50	36	14(38.89%)	22(61.11%)	0.943
**Stage**				
Early (T1+T2)	24	7(29.17%)	17(70.83%)	
Late (T3)	53	23(43.40%)	30(56.6%)	0.236
**Grade**				
Low (1&2)	27	16(59.26%)	11(40.74%)	
High (3)	36	11(30.56%)	25(69.44%)	0.023*
**Histology**				
clear cell	17	2(11.76%)	15(88.24%)	
others	61	28(45.90%)	33(54.10%)	0.011*
**Recurrance**				
+	35	13(37.14%)	22(62.86%)	
−	34	15(44.12%)	19(55.88%)	0.555

**Table 2:** Clinicopathological analysis of PITX2 expression in ovarian cancer tissue array (OVC1021, Pantomics, Inc.).

		PITX2 expression		
Characteristics	Total	< = 4 folds	>4 folds	*P-value*
**All cases**	97	32(33.0%)	65(67.0%)	
**Stage**				
Early (T1+T2)	73	21(28.8%)	52(71.2%)	
Late (T3)	24	11(45.8%)	13(54.2%)	0.139
**Grade**				
Low (1&2)	50	27(54.0%)	23(46.0%)	
High (3)	46	4(8.7%)	42(91.3%)	<0.001*
**Metastasis** (Regional Lymph node+Distant)				
Absence	73	21(28.8%)	11(71.2%)	
Presence	24	11(45.8%)	13(54.2%)	0.139

### PITX2 increases cell growth in ovarian cancer cells

**Figure 2:**
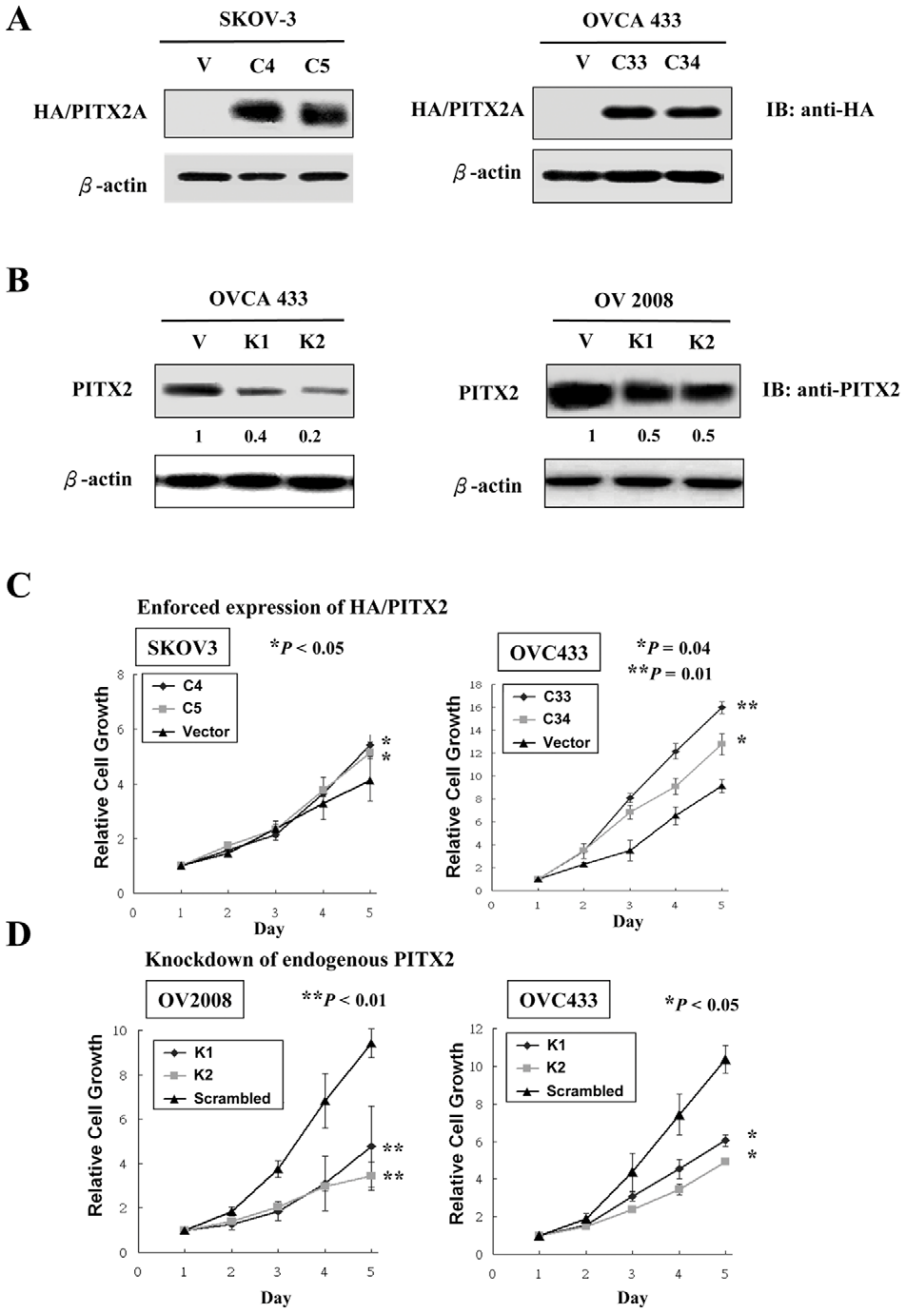
PITX2 promotes ovarian cancer cell growth. (**A**) PITX2A stable expressing clones were established in SKOV3 and OVCA433 cells. Western blot analysis using anti-HA antibody showed the expression levels of HA-tagged PITX2A in C4 and C5 clones of SKOV-3, and C33 and C34 clones of OVCA 433. β-actin was used as loading control. (**B**) Western blot analysis using anti-PITX2 antibody showed the reduced expressions of endogenous PITX2 in stable knockdown clones generated by 2 shRNA constructs (K1 and K2). Scrambled is the non-specific shRNA control. The numerical units represent the relative expressions of PITX2 reduction in each stable clone as compared with the scrambled controls. β-actin was used as loading control. (**C**) Ectopic expression of PITX2A stimulated cell proliferation in ovarian cancer cells. Both C4 and C5 of SKOV-3 cells demonstrated 1.5- fold increase of cell growth (* *P*<0.05), C33 and C34 of OVCA 433 cells had 3- fold increase of cell growth (** *P*<0.01) as compared with their vector control. (**D**) Depletion of endogenous PITX2 reduced cell proliferation in ovarian cancer cells. A 3- to 4- fold of reduction on cell viability in both knockdown clones (K1 and K2) of OV2008 cells (** *P*<0.01) and 2- to 3- fold decrease on cell proliferation in knockdown clones (K1 and K2) of OVCA 433 cells (* *P*<0.05) were observed.

Given that the upregulated PITX2 was associated with high-grade ovarian cancer, PITX2 may possess oncogenic functions in mediating aggressive phenotype in ovarian cancer cells. To test this notion, we first generated PITX2 stable expressing clones from two high-grade ovarian cancer cell lines (SKOV3 and OVCA433) (Figure 2A). On the other hand, another high-grade ovarian cancer cell lines with PITX2-high expression (OV2008), as well as OVCA433 were chosen for stable knockdown of PITX2 using vector-based RNAi approach. Four shRNA constructs targeting at different sites of PITX2 open-reading frame were transiently transfected into the cells respectively and two of them (K1 and K2) yielded 50–70% reduction of endogenous PITX2 was chosen for stable transfection (Figure 2B). By XTT cell proliferation assay, the relative growth rate of the PITX2 stably expressing clones in SKOV3 and OVCA433 cells was significantly higher (1.5- to 3-fold) than their vector controls (Figure 2C). In reverse, depletion of PITX2 in OV2008 and OVCA433 cells significantly impaired their cell proliferation rate by 2- to 3-fold as compared with their scrambled controls (Figure 2D).

**Figure 3:**
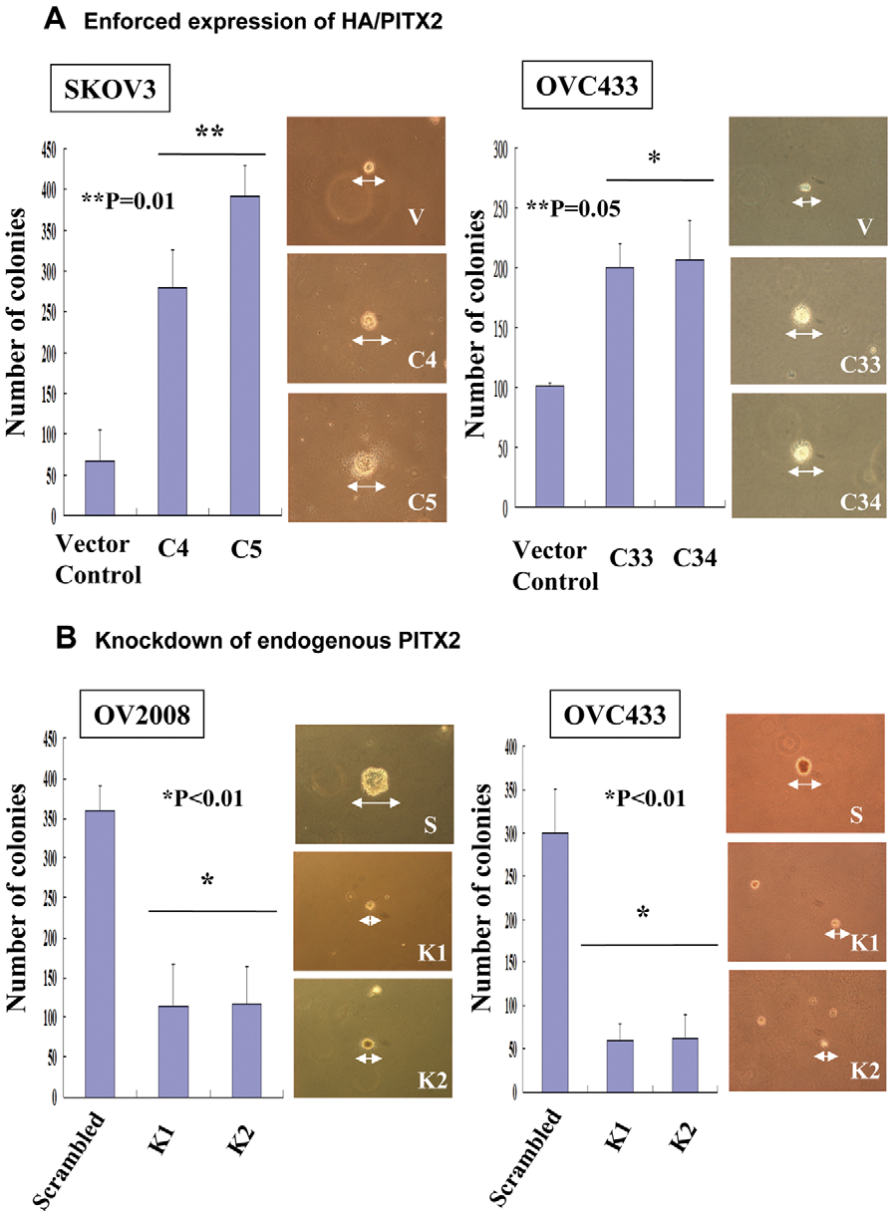
PITX2 enhances anchorage-independent growth ability of ovarian cancer cells. (**A**)Enforced expression of PITX2A enhanced anchorage independent growth ability of ovarian cancer cells. The bar chart shows that PITX2A stably expressing clones (C33 and C34) of OVCA 433 (**P = 0.05) and (C4 and C5) of SKOV-3 (*P = 0.01) had increased number of colonies in soft agar compared with their vector controls. Representative pictures show larger colony size in PITX2A stable expressing clones under microscopy. (**B**)Depletion of PITX2 by shRNA inhibited anchorage independent growth ability of ovarian cancer cells. The number of colonies in PITX2stable knockdown clones (K1 and K2) of OVCA 433 and OV2008 showed significantly reduction as compared with the scrambled controls (* *P*<0.01). Representative pictures shows that the reduced colony size of PITX2stableknockdown clones and their scrambled controls. The above experiment was repeated at least three times independently and the data was calculated with mean ± SD.

Furthermore, enforced expression of PITX2 increased not only the size but also the number of colonies in OVCA433 and SKOV3 cells by 2-fold (*P*<0.05) and 8-fold (P<0.01) respectively (Figure 3A). In contrast, depletion of PITX2 reduced both size and number of colonies in OV2008 and OVCA433 cells by 2.6-fold to 5-fold (*P*<0.01) respectively as compared with their scrambled control (Figure 3B). Collectively, these data suggest that the upregulation of PITX2 could promote cell growth of ovarian cancer cells and support the oncogenic roles of PITX2 in high-grade ovarian tumor.

### PITX2 enhances cell migration and invasion of ovarian cancer cells

**Figure 4:**
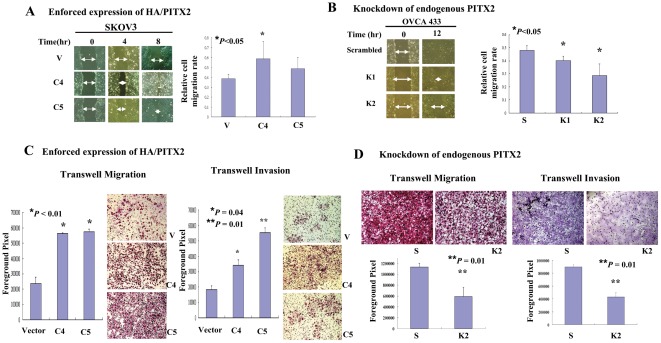
PITX2 promotes ovarian cancer cell migration and invasion. (**A**) Wound healing assay showed that PITX2 stably expressing cells (C4 of SKOV-3) exhibited faster wound closure rate than the vector control in the time course of 8 hr (**P<0.05). (**B**) PITX2 stable knockdown clones (K1 and K2 of OVCA 433) showed significant reduction on wound closure rate compared with vector control in the time course of 12 hrs (*P<0.05). The arrows indicate the width of wound and the relative cell migration rate is expressed as relative width of the wounds/time. The assay was repeated three times independently. (**C**) Transwell migration and invasion assay demonstrated that PITX2 stably expressing clones in SKOV3 (C4 and C5) cells migrate faster through the membrane (*P<0.01) and invade faster through the matrigel (C4, *P = 0.04 and C5, **P = 0.01) compared with the vector control. (**D**) PITX2 stable knockdown clone (K2 of OVCA 433) exhibited remarkable reduction in cell penetration through the membranes and cell invasiveness compared with the scrambled controls (**P = 0.01). Three views were randomly picked in each inserts and the numbers of invaded cell were counted. The results of three independent experiments were plotted with error bar.

Previous studies have shown that PITX2 could trigger neuronal cell migration during the development of mouse hypothalamus [16], indicating that PITX2 possesses cell migratory promoting capacity. Here, we attempted to investigate whether PITX2 confers a role in promoting cell migration and invasion of ovarian cancer cells. We first conducted wound healing assay to examine whether PITX2 could promote cell migration. Upon treatment of Mitomycin C to exclude the factor of increased cell growth, we observed that enforced expression of PITX2in SKOV3 cells exhibited a faster wound closure rate than the vector control (Figure 4A). Conversely, knockdown of endogenous PITX2 in OVCA433 cells remarkably reduced the cell migration rate observed in wound healing assay (*P*<0.05) (Figure 4B). Furthermore, by Transwell migration and invasion assays, we demonstrated there were 2- to 3-fold (*P*<0.01)and 0.8- to 1.5-fold (*P* = 0.04 and 0.01) increase in cell migration rate and invasion rate respectively in PITX2 stable expressing clones (C4 and C5) as compared with the vector control of SKOV3 cells (Figure 4C). In contrast, the numbers of cell penetrated through membrane in Transwell migration assay and invaded through matrigel in Transwell invasion assay were significantly reduced in PITX2 stable knockdown clones (K1 and K2) as compared with scrambled control of OVCA433 (*P*<0.01) (Figure 4D). These data suggest that PITX2 possesses capacity in promoting cell migration and invasion in ovarian cancer cells.

### PITX2 regulates Cyclin-D1 and C-myc in ovarian cancer cells

**Figure 5:**
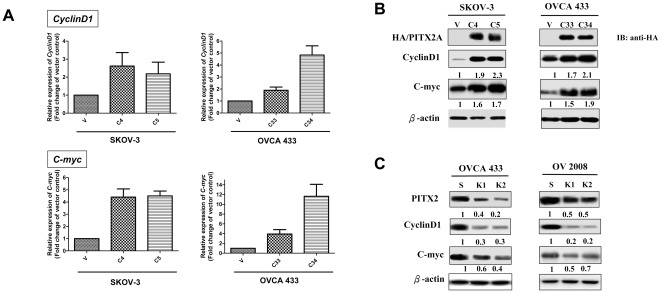
PITX2 elevates the expressions of *Cyclin-D1* and *C-myc* in ovarian cancer cells. (**A**) Q-PCR analysis revealed that the levels of *Cyclin-D1* and *C-myc* were elevated in PITX2A stably expressing clones (C4, C5 of SKOV-3 and C33, C34 of OVCA 433). (**B**)Western blot analysis showed that the protein levels of Cyclin-D1 and C-myc were increased by PITX2 in PITX2A stably expressing clones (C4, C5 of SKOV-3 and C33, C34 of OVCA 433). (**C**) Knockdown of PITX2 significantly reduced the levels of Cyclin-D1 and C-myc in PITX2 knockdown stable clones (C6, C8 of OVCA 433 and C1, C4 of OV2008). The levels of HA-tagged PITX2A were detected using anti-HA antibody, while endogenous PITX2 expression was examined by anti-PITX2 antibody, β-actin was used as loading control. The numerical value under each panel represents the relative expression of Cyclin-D1 and C-myc to their vector controls.

Cyclin-D1 and C-myc are two crucial regulators in promoting cell proliferation [17]. To address whether PITX2 regulates these genes in promoting cell growth of ovarian cancer cells, we firstly performed Q-PCR analysis and showed that there was 2–12 folds increase in expressions of *Cyclin-D1* and *C-myc* in PITX2 stably expressing ovarian cancer cells (Figure 5A). Additionally, Western blot analysis also revealed that both Cyclin-D1 and C-myc elevated at least 60% in PITX2 stably expressing ovarian cancer cells (Figure 5B). Conversely, knockdown of PITX2 remarkably reduced the expressions of Cyclin-D1 and C-myc at least 20% compared to their scrambled controls in OVCA 433 and OV2008 cells (Figure 5C).These findings indicate that PITX2 could upregulate Cyclin-D1 and C-myc in promoting cell growth of ovarian cancer cells.

### PITX2 promotes *in vivo* tumor growth of ovarian cancer cells

**Figure 6:**
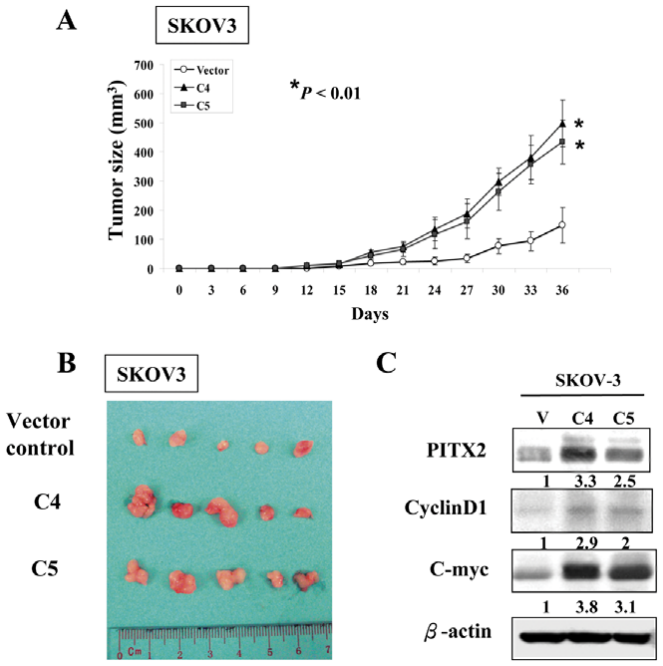
PITX2 promotes the tumor growth in nude mice. (**A**) PITX2 stably expressing clones in SKOV3 (C4 and C5) showed faster in the tumor growth in nude mice as compared with the vector control. The tumor size was represented by the mean ± SE of five mice and was measured for 36 days.*, *P*<0.01, significantly different from vector control group. (**B**) Photograph illustrates the dissected tumors taken from nude mice subcutaneously injected by two PITX2 stable expressing clones in SKOV3 (C4 and C5) and the vector control on Day 36. (**C**) Western blot analysis showed the expressions of PITX2, Cyclin-D1 and C-myc from the mice subcutaneous tumor tissues. The numerical value under each panel represents the relative expression of Cyclin-D1 and C-myc to their vector controls.

Apart from *in vitro* tumorigenic studies on PITX2, we attempted to determine whether overexpression of PITX2 could enhance tumor growth ability in a xenograft mouse model. Two PITX2 stable expressing clones (C4 and C5) and a vector control of SKOV3 were inoculated subcutaneously in nude mice. After 36 days, we observed that both stable clones with PITX2 overexpression had 2.5–3 fold increase intumor growth rate compared to the vector control (*P*<0.01) (Figure 6A & 6B). Western blot analysis on the dissected tumor tissues collected on Day 36 showed that both PITX2 stable expressing clones (C4 and C5) of SKOV3 expressed high levels of PITX2 and concomitant with elevated expressions of Cyclin-D1 and C-myc (Figure 6C). This result is consistent with the *in vitro*tumorigenic data and further supports that PITX2 could promote tumor growth through upregulation of Cyclin-D1 and C-myc.

### PITX2 is regulated by Wnt/β-catenin independently in ovarian cancer cells

Previous reports have documented that PITX2 is the downstream mediator of Wnt/β-catenin pathway [12], [18]. Therefore, we were of interested to examine whether the upregulation of PITX2 is due to the activated Wnt/β-catenin. Upon treatment of GSK3β inhibitor (lithium chloride), we found that the Wnt/β-catenin activity was activated through the increased expression of β-catenin in a dose-dependent manner in OVCA433 and A2780cp cells. However, there was only slight or even no change of PITX2 expression in both cell lines (Figure S2A). Likewise, upon treatment of Wnt3A condition medium, both A2780cp and OVCA 433 cells showed unobvious response to the increased Wnt/β-catenin activity (Figure S2B). These findings indicate that the upregulation of PITX2 expression in ovarian cancer cells is not mainly regulated by Wnt/β-catenin signaling but maybe by other molecular mechanisms.

## Discussion

Tumor progression is a multi-step process which advances cancer to be a more malignant and aggressive phenotype [19]. High-grade tumor represents a more advanced progression which possesses higher cell proliferative and invasiveness capacities [20]. In this study, we firstly demonstrated that PITX2 was frequently upregulated in ovarian cancers particularly in high-grade and clear cell subtype of ovarian cancer using Q-PCR, Western blot and IHC analyses. More importantly, we addressed the oncogenic role of PITX2 possesses in cell proliferation, anchorage-independent growth ability, cell migration/invasion, as well as tumor growth in a tumor xenograft mice model.

According to FIGO grading classification, high-grade ovarian tumor cells are usually poorly histological differentiated [20], grow faster and highly metastatic [7]. In addition, prognosis of high-grade ovarian tumor is poor thereafter it often associates with poor survival rate [21], [22].The clear cell subtype ovarian cancer accounts for approximately 6% of all epithelial ovarian tumors and most cases of this subtype are high-grade tumor exhibiting an aggressive phenotype [3], [22], [23]. Our study showed that both mRNA and protein levels of PITX2 was frequently upregulated in ovarian cancer particularly in the high-grade and clear cell subtypes, indicating that PITX2may play an important role in driving aggressive phenotypes in ovarian cancer.

PITX2 is a homeodomain transcription factor which plays an essential role in embryonic development such as liver, hematopoiesis, gonads, and neuronal differentiation etc [24], [25], [26]. Recently, there have been increasing reports showing that PITX2 is overexpressed in human cancers such as nonfunctional pituitary adenomas [10], Wilms tumor [27] and node-positive colorectal cancer [11]. However, the functional roles of PITX2 in human cancers such as ovarian cancer remain unknown. In order to unveil the functional roles of PITX2 in ovarian cancer, we generated gain or loss of function of PITX2 in high-grade ovarian cancer cell models and performed a series of *in vitro* and *in vivo* tumorigenic assays with regard to the effect of PITX2 in tumor cell growth and metastasis. Our study demonstrated that enforced expression of PITX2 could significantly enhance cell proliferation, anchorage independent growth ability, cell migration/invasion and tumor growth of ovarian cancer cells in mouse xenograft tumor model. Conversely, depletion of PITX2 by shRNA impaired the above tumorigenic phenotypes in high-grade ovarian cancer cell models. Taken together, our findings suggest that the oncogenic functions possessed by PITX2 are exclusively found in high-grade ovarian cancer and are consistent with the clinicopathological analysis of that the overexpressed PITX2 is closely correlated with high-grade ovarian cancer.

Previous studies have showed that PITX2 elevates cell cycle regulatory factors such as Cyclin-D1, -D2 and C-myc promoting cell-type specific proliferation in murine pituitary and myoblast cell lines [18], [28], [29], [30]. In addition, biochemical studies depicted that PITX2 binding sites are located along the promoter regions of *Cyclin-D1*, -*D2* and *C-myc*, suggesting that these genes are direct transcription targets of PITX2 [18], [28], [29], [30], [31], [32]. Furthermore, activation of oncogenes such as Cyclin-D1 and C-myc could enhance anchorage-independent growth in mouse mammary epithelial cells on and tumor growth in severe combined immunodeficiency (SCID) mice [17]. These suggest that the upregulation of Cyclin-D1 and C-myc contributes to more aggressive features of tumors including metastasis. We thus speculated that PITX2 enhanced cell proliferation, anchorage-independent growth ability and *in vivo* tumor growth through upregulation of these genes. As expected, our data clearly showed that the expressions of both Cyclin-D1 and C-myc were upregulated by PITX2even in tumor tissues obtained from tumor xenograft mice, whereasPITX2 depleted ovarian cancer cells exhibited reduction in the expressions of Cyclin-D1 and C-myc. Collectively, these findings manifest PITX2 acts as a transcription factor activating downstream oncogenes in mediating tumor progression.

Previous studies have pointed out that PITX2 is one of the downstream effectors of Wnt/β-catenin pathway [18], [28], [31]. However, our finding could not show any relationship between Wnt/β-catenin signaling and PITX2 expression in ovarian cancer cells. We showed that there was no significant change of PITX2 expression upon activation of Wnt/β-catenin activity in ovarian cancer cell lines, suggesting that the aberrant upregulation of PITX2 may be regulated in-dependently by canonical β-catenin pathway in ovarian cancer cells. Therefore we speculated that the upregulation of PITX2 in ovarian cancer might be due to other genetics alterations. Further investigation in molecular mechanism leading to PITX2 overexpression is warranted.

In conclusion, our findings suggest that PITX2 plays an important role in ovarian cancer progression by promoting tumor growth and migration/invasion in aggressive high-grade ovarian cancer.

## Supporting Information

Figure S1 (**A**) Western blot analysis showed the PITX2 was upregulated in TOV21G clear cell subtype cell line as copared with an immortalized normal fallopian tube epithelial cell line OE-E6/E7. (B) Immunohistochemical study on a commercial tissue array 9OVC481, Pantomics) showed that increased expression of PITX2 was observed in high-grade serous cystadenocarcinoma and undifferentiated carcinoma as compared with their paired uninvolved normal ovaries. Magnification: 20×.
(TIF)

Figure S2 **The expression of PITX2 is not altered by Wnt/β-catenin activity in ovarian cancer cells.** Western blot analysis showed that activation of β-catenin either by (**A**) Lithium chloride, or (**B**) treatment of Wnt3a media did not elevate PITX2 levels in A2780cp and OVCA 433 cells. The numerical value under each panel represents the relative expression of PITX2 compared with the untreated control.
(TIF)
