# Supplementary figures and images for: Increased Expression of PITX2 Transcription Factor Contributes to Ovarian Cancer Progression
Source: PLoS One. 2012 May 15;7(5):e37076. doi: 10.1371/journal.pone.0037076 (PMC3352869; doi:10.1371/journal.pone.0037076)

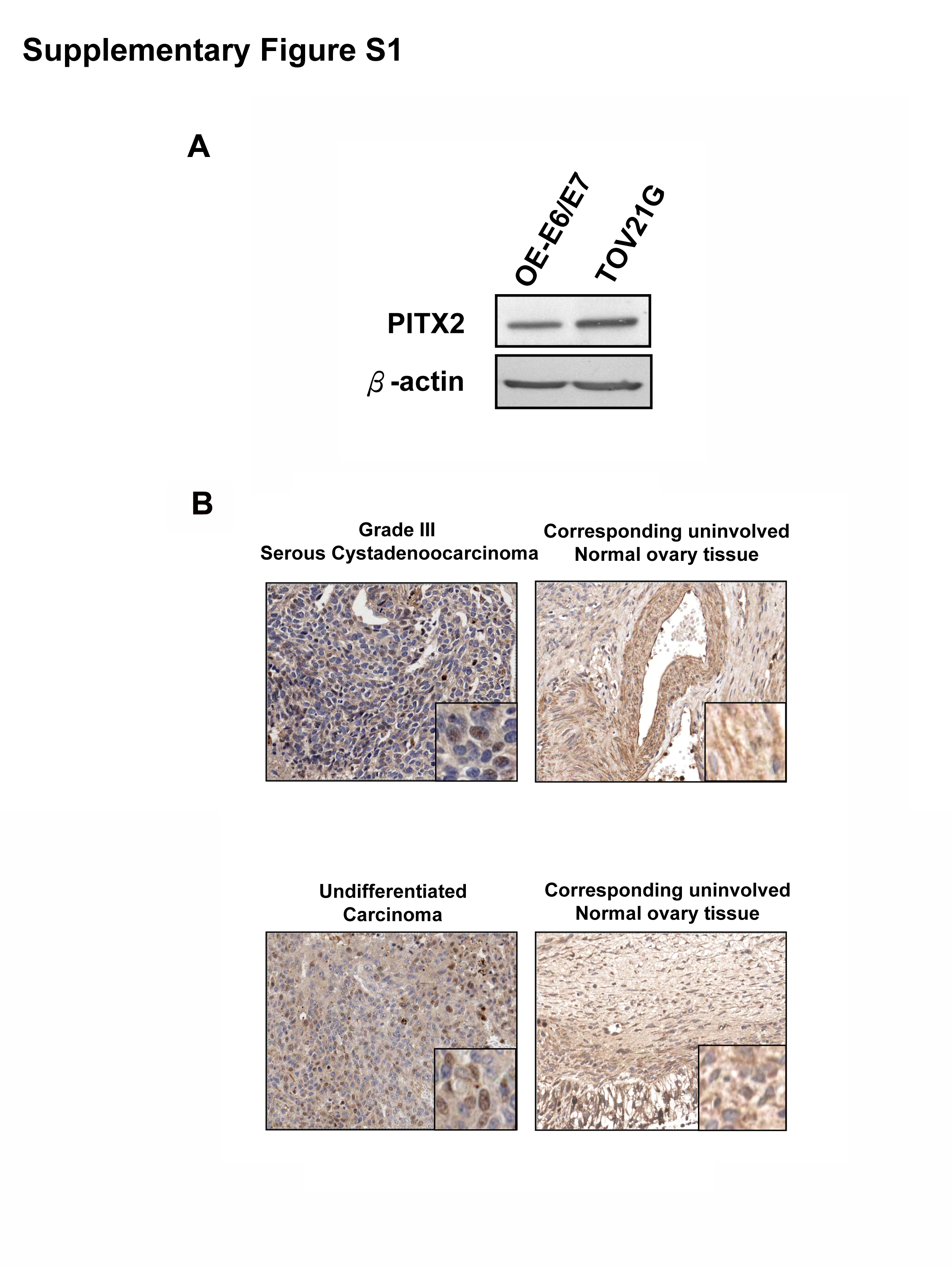

Supplement: Figure S1 — (A) Western blot analysis showed the PITX2 was upregulated in TOV21G clear cell subtype cell line as copared with an immortalized normal fallopian tube epithelial cell line OE-E6/E7. (B) Immunohistochemical study on a commercial tissue array 9OVC481, Pantomics) showed that increased expression of PITX2 was observed in high-grade serous cystadenocarcinoma and undifferentiated carcinoma as compared with their paired uninvolved normal ovaries. Magnification: 20×. (TIF) [file pone.0037076.s001.tif]

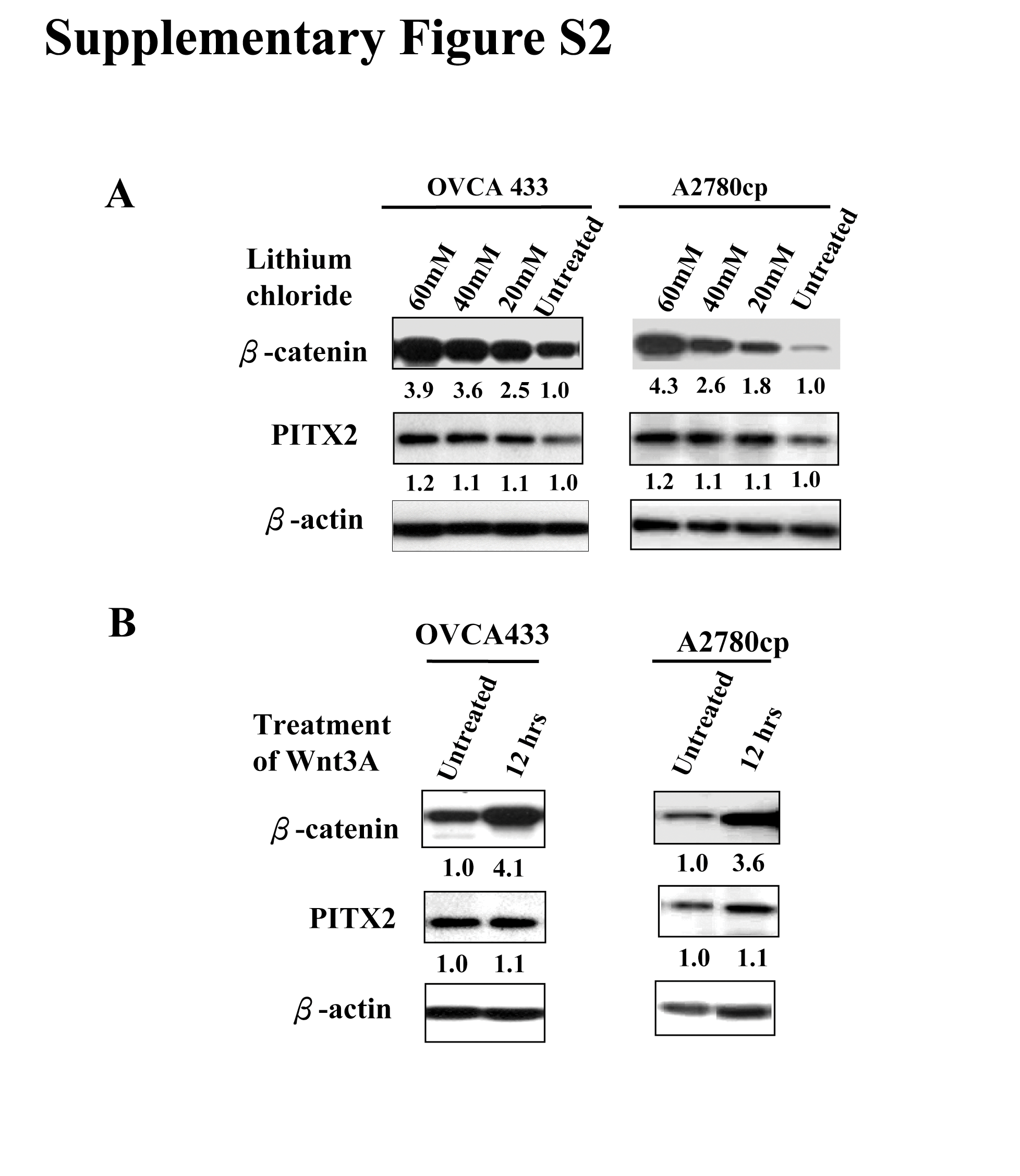

Supplement: Figure S2 — The expression of PITX2 is not altered by Wnt/β-catenin activity in ovarian cancer cells. Western blot analysis showed that activation of β-catenin either by (A) Lithium chloride, or (B) treatment of Wnt3a media did not elevate PITX2 levels in A2780cp and OVCA 433 cells. The numerical value under each panel represents the relative expression of PITX2 compared with the untreated control. (TIF) [file pone.0037076.s002.tif]
